# Socioeconomic and clinical risk factors associated with moderate intensity physical activity before and during the COVID-19 pandemic

**DOI:** 10.1016/j.pmedr.2023.102555

**Published:** 2023-12-10

**Authors:** David Adzrago, Saanie Sulley, Cameron K. Ormiston, Faustine Williams

**Affiliations:** a Division of Intramural Research, National Institute on Minority Health and Health Disparities,National Institutes of Health, Bethesda, MD, USA; b National Healthy Start Association, Washington, DC, USA

**Keywords:** Physical activity, Moderate intensity physical activity, COVID-19 pandemic, Mental health, Anxiety, Depression, Obesity, Disparities, Cancer diagnosis, Socioeconomic

## Abstract

Although moderate intensity physical activity (MIPA) improves general mental health, morbidity, and mortality, the COVID-19 pandemic may have adversely impacted individuals’ ability to engage in MIPA. We examined the extent of socioeconomic factors, body mass index, anxiety/depression, and cancer diagnosis associated with MIPA before and during the COVID-19 pandemic. Multivariable logistic regression models were conducted on 4,551 U.S. adults aged 18–64 years using nationally representative samples from the 2019 and 2020 Health Information National Trends Survey datasets. The prevalence of MIPA before the pandemic was higher (77.07%) than during the pandemic (76.21%). Before the COVID-19 pandemic, lower odds of MIPA were observed for individuals with fair/poor health (OR = 0.24, 95% CI = 0.27, 0.63), obesity (OR = 0.51, 95% CI = 0.33, 0.79), anxiety/depression (OR = 0.60, 95% CI = 0.42, 0.85), or a cancer diagnosis (OR = 0.56, 95% CI = 0.32, 0.98). During the pandemic, lower odds were observed among those with obesity (OR = 0.44, 95% CI = 0.28, 0.70), anxiety/depression (OR = 0.61, 95% CI = 0.43, 0.86), less than high school education (OR = 0.33, 95% CI = 0.16, 0.70), or family income of $20,000 - $34,999 (OR = 0.42, 95% CI = 0.24, 0.74). There was a decline in MIPA during the pandemic, with certain subgroups, such as individuals of lower socioeconomic and physical and mental health status, less likely to engage in MIPA. This study highlights the need for concerted physical activity educational strategies aimed at improving access to and utilization of MIPA within subgroups to reduce MIPA disparities, particularly among disadvantaged groups during pandemics.

## Highlights

• COVID-19 pandemic affects moderate intensity physical activity (MIPA) in U.S. adults.
• MIPA was lower during the pandemic than before the pandemic.
• MIPA declined among older adult, male, and lesbian/gay/bisexual individuals.
• MIPA declined among Hispanics/Asians/multiracial and those in U.S. South/Northeast.
• Underweight/obese persons and those with severe anxiety/depression had decline MIPA.

## 1 Introduction

Physical activity is associated with health benefits, including improvement in general health status, sleep quality, brain health and mental health (e.g., depression and anxiety), morbidity, mortality, weight management, and reduced risk of certain chronic diseases (Kannel and Sorlie, Aug 1979, Warburton et al., 2006, Warburton and Bredin, 2017, Blair and Connelly, 1996, Wannamethee and Shaper, 2001, Hupin et al., 2015, Kline et al., 2021, Biddle and Asare, 2011, Tyson et al., 2010, Stubbs et al., 2017). Moreover, physical activity has been shown to decrease the potential hospitalizations associated with chronic conditions such as obesity, hypertension, diabetes, and heart failure (Koo et al., 2017, Vasconcelos et al., 2015, Lu et al., 2019, Nelson et al., 1986, Knowles et al., 2013). Before the COVID-19 pandemic, it was estimated that 36% of the adult population in the United States (U.S.) performed no leisure-time physical activity, and 22% met combined aerobic and muscle-strengthening exercise requirements according to 2018 federal physical activity guidelines (Piercy and Troiano, 2018). The disparities in physical activities might be exacerbated by the COVID-19 pandemic, given the drastic changes in lifestyles and the limited use of recreational facilities during this period (Puccinelli et al., 2021, Stockwell et al., 2021).

Previous studies have documented a reduction in exercise activities during the COVID-19 pandemic and associated lack of physical activity with severe COVID-19 outcomes (Puccinelli et al., 2021, Stockwell et al., 2021, Després, Aug 2021, Rahmati et al., 2022, Sallis et al., 2021, Watson et al., 2021). During the COVID-19 pandemic, some community spaces or places (e.g., gyms, parks, recreational venues, and fitness facilities) for physical activity were deemed as areas of high risk COVID-19 exposure and were, therefore, shut down by state and local authorities across the U.S. to reduce the spread of the virus (Kates et al., 2020). This decision, coupled with quarantine and stay-at-home orders, had direct and indirect impacts on people’s physical activity levels and access to recreational facilities to exercise (Kates et al., 2020). Additionally, quarantine and closure of fitness centers across the nation may have had adverse effects on people’s mental and physical health, particularly among those with pre-existing mental health conditions, those who smoke, and other persons with increased COVID-19 mortality and morbidity risk (CDC COVID-19 Response Team, 2020, Mayo Clinic Staff, 2023). Furthermore, studies have also reported that management and treatment for chronic conditions, sleep, and anxiety were negatively impacted during the pandemic (Chudasama et al., 2020, Morin et al., 2021, Singh et al., 2022, Knowland et al., 2022). With many shifting to virtual and work-from-home jobs, the amount of time spent sitting down—a known contributor to poor health—was high among U.S. adults, with over 40% of adults sitting more than 8 hours per day (Meyer et al., 2020). Moreover, existing disparities in physical activity may have also increased due to the disproportionate impact of the pandemic and its downstream effects on marginalized groups such as racial/ethnic minorities (Webb Hooper et al., 2020). For instance, a trend analysis examining physical activity guidelines adherence by race/ethnicity revealed that non-Hispanic White Americans exercised more than racial/ethnic minority groups (Watson et al., 2021).

Given the importance of physical activity in improving individual’s health, it is imperative to understand how the COVID-19 pandemic impacted physical activity among subgroups of the U.S. adult population. This study investigates differences in moderate physical activity among various subgroups of U.S. adults considering socioeconomic factors, body mass index (BMI), anxiety/depression, and cancer diagnosis before and during the COVID-19 pandemic. Specifically, this study (1) estimates the prevalence of moderate intensity physical activity by socioeconomic factors, health behaviors, and mental and physical health status, and (2) examines the associations of moderate intensity physical activity with socioeconomic factors, health behaviors, and mental and physical health status before and during COVID-19 pandemic. By examining these differences in moderate physical activities before and during the COVID-19 pandemic, we can gain valuable insights into effective strategies to promote and sustain physical activity levels during strict public health emergencies, ultimately enhancing individuals’ health. Additionally, this research can help inform public health policies and interventions aimed at increasing moderate physical activity levels among the U.S. population and subgroups during pandemics.

## 2 Methods

### 2.1 Study population and data

The 2019 and 2020 Health Information National Trends Survey (HINTS 5, cycles 3 and 4) de-identified public-use data from January through April 2019 and February through June 2020, respectively were utilized for this study (Finney Rutten et al., 2020, Westat, 2020). HINTS is a nationally representative annual cross-sectional survey of the non-institutionalized adult population of the U.S. and evaluates their health-related information and behaviors (e.g., moderate intensity physical activity, anxiety/depression, smoking, and cancer diagnosis). Stratified sampling was used to sample the participants based on minority concentration (large versus small) of the population. A total sample of 9,303 adults aged 18 years or more were assessed in HINTS 5 cycles 3 (n = 5,438) and 4 (n = 3,865). A total of 5,933 adults aged 18–64 years were assessed in 2019 and 2020 surveys, but our analytical sample included 4,551 adults aged 18–64 years with complete cases. Our analysis did not include older adults aged 65 years or older because they have different physical activity recommendations, according to the Centers for Disease Control and Prevention (National Center for Chronic Disease Prevention and Health Promotion). All analyses samples are weighted by the household size and oversampling was applied to minority samples. Given that HINTS data have been de-identified and publicly available, an Institutional Review Board approval was not required to access the data for our study.

### 2.2 Measures

#### 2.2.1 Dependent variable

Moderate intensity physical activity status was assessed in the HINTS 5 surveys. The participants were asked, “In a typical week, how many days do you do any physical activity or exercise of at least moderate intensity?” with possible responses comprising 0, 1, 2, 3, 4, 5, 6, or 7 days. The possible responses of this variable were dichotomized into no moderate intensity physical activity (response = 0 days) and at least one day of moderate intensity physical activity (response = 1, 2, 3, 4, 5, 6, or 7 days).

#### 2.2.2 Independent variables

We examined self-reported age in years (18–25, 26–34, 35–49, 50–64), sex (male or female), and race/ethnicity (Hispanic, non-Hispanic Asian, non-Hispanic Black/African American, non-Hispanic other racial/ethnic group, and non-Hispanic White), sexual orientation (bisexual, heterosexual, and lesbian/gay), level of education completed (<high school, high school graduate, some college, or college graduate or higher), marital status (married/living as married/living with a romantic partner, separated/divorced, single/never been married, or widowed), total annual family income (<$20,000, $20,000-$34,999, $35,000-$49,999, $50,000-$74,999, or ≥$75,000), health insurance (no or yes), general health status (excellent very good, good, fair, or poor), U.S. census region (Midwest, Northeast, West, or South), and cancer diagnosis status (no or yes). In addition, we examined BMI (underweight [<18.5], healthy/normal weight [18.5–24.9], overweight [25.0–29.9], obese [≥30.0]), current anxiety/depression status (minimal/negative, mild, moderate, and severe**)**, and cigarette smoking status (non-smoker, former smoker, and current smoker). The current anxiety/depression categories were derived from Patient Health Questionnaire-4 (PHQ-4) with a total score of 0–12, where scores are rated as minimal/negative = 0–2, mild = 3–5, moderate = 6–8, and severe = 9–12 (Kroenke et al., 2009, Löwe et al., 2010). Due to small samples/data within some subgroups, we recategorized the categories of the following variables into smaller categories: general health status (excellent/very good/good or fair/poor), sexual orientation (heterosexual or lesbian/gay/bisexual), and current anxiety/depression (minimal/no anxiety/depression or mild/moderate/severe anxiety/depression).

### 2.3 Statistical analyses

All the analyses were weighted by sampling weight and replicate weight, according to recommendations described by the HINTS, to offset non-response bias and obtain nationally representative estimates (Westat, 2020). By surveys before and during the COVID-19 pandemic, we computed the prevalence of moderate intensity physical activity by sociodemographic characteristics and status of BMI, anxiety/depression, cigarette smoking, and cancer diagnosis (see Table 1). We also computed bivariate χ2 analysis to assess the association between moderate intensity physical activity and sociodemographic characteristics, the status of BMI, anxiety/depression, cigarette smoking, and cancer diagnosis in the surveys before and during the COVID-19 pandemic. Additionally, we conducted multivariable logistic regression to estimate odds ratios (ORs) and 95% confidence intervals (95% CIs) for the association of moderate intensity physical activity in U.S. adults aged 18–64 years with sociodemographic characteristics and status of BMI, anxiety/depression, cigarette smoking, and cancer diagnosis before (Model 1) and during (Model 2) COVID-19 pandemic. The multivariable analysis was conducted using a complete case analysis. STATA/SE 16.1 (Stata, 2019) was used to perform all analyses and statistically significant results were reported at *p*-value < 0.05 and 2-sided.

**Table 1:** Demographic characteristics of U.S. adults aged 18–64 years before and after the COVID-19 pandemic and association with moderate intensity physical activity (n = 4,551), HINTS 2019–2020.

	**Before the COVID-19 pandemic**			**During the COVID-19 pandemic**		
	**Physical activity intensity**			**Physical activity intensity**		
	**Total (n)**	**n (%)**	***P***	**Total (n)**	**n (%)**	***P***
**Characteristics**	n = 2,653	2,078 (77.07)		n = 1,898	1,470 (76.21)	
**Age (Years)**			0.277			0.193
18–25	146 (13.64)	117 (76.20)		125 (17.25)	99 (76.34)	
26–34	420 (16.13)	361 (84.42)		306 (17.71)	265 (83.66)	
35–49	823 (32.33)	637 (75.31)		590 (32.31)	461 (75.41)	
50–64	1,264 (37.90)	963 (75.76)		877 (32.73)	645 (72.89)	
**Sex**			0.776			0.917
Female	1,535 (48.66)	1,179 (76.66)		1,102 (49.25)	844 (76.37)	
Male	1,118 (51.34)	899 (77.45		796 (50.75)	626 (76.06)	
**Race/Ethnicity**			0.146			0.171
Non-Hispanic White	1,592 (61.31)	1,264 (77.54)		1,115 (62.21)	889 (78.88)	
Non-Hispanic Black	373 (11.87)	279 (70.85)		238 (10.51)	170 (74.08)	
Hispanic	441 (17.40)	347 (75.61)		374 (18.34)	277 (71.93)	
Non-Hispanic Asian	136 (6.03)	107 (83.64)		93 (5.46)	71 (72.63)	
Non-Hispanic other	111 (3.39)	81 (86.08)		78 (3.49)	63 (63.27)	
**Sexual orientation**			0.116			0.886
Heterosexual	2,515 (94.96)	1,965 (76.63		1,779 (93.71)	1,383 (76.28)	
Lesbian/gay/bisexual	138 (5.04)	113 (85.23)		119 (6.29)	87 (75.24)	
**Marital status**			0.181			0.393
Single/never married	569 (34.57)	442 (75.64)		420 (35.67)	334 (77.12)	
Married/living as married	1,593 (57.03)	1,264 (78.80)		1,116 (54.47)	869 (76.35)	
Divorced/separated	427 (7.50)	330 (72.90)		299 (8.62)	218 (70.25)	
Widowed	64 (0.90)	42 (56.92)		63 (1.23)	49 (85.63)	
**Level of education completed**			<0.001			<0.001
Less than high school	108 (5.69)	69 (62.45)		98 (6.15)	52 (52.30)	
High school graduate	361 (20.02)	255 (71.59)		286 (19.84)	200 (70.27)	
Some college	775 (41.99)	575 (74.85)		531 (39.87)	392 (76.00)	
College graduate/higher	1,409 (32.30)	1,179 (85.92)		983 (34.14)	826 (84.23)	
**Total annual family income**			<0.001			<0.001
<$20,000	377 (16.19)	255 (64.39)		250 (12.30)	167 (68.43)	
$20,000 - $34,999	245 (8.46)	176 (71.28)		197 (10.01)	132 (61.13)	
$35,000 - $49,999	295 (12.96)	224 (73.27)		219 (11.04)	160 (73.93)	
$50,000 - $74,999	482 (17.49)	385 (80.41)		325 (18.02)	253 (73.46)	
>$74,999	1,254 (44.90)	1,038 (82.53)		907 (48.63)	758 (82.82)	
**Health insurance**			0.026			0.162
No	199 (10.10)	141 (67.82)		140 (10.56)	100 (68.95)	
Yes	2,454 (89.90)	1,937 (78.11)		1,758 (89.44)	1,370 (77.07)	
**General health status**			<0.001			0.003
Excellent/very good/good	2,300 (85.75)	1,878 (81.12)		1,680 (88.71)	1,344 (78.10)	
Fair/poor	353 (14.25)	200 (52.69)		218 (11.29)	126 (61.36)	
**U.S. census region**			0.797			0.003
Northeast	374 (16.97)	292 (74.61)		283 (17.67)	213 (64.30)	
Midwest	469 (21.10)	360 (78.26)		319 (20.35)	259 (83.14)	
West	694 (24.02)	554 (76.16)		479 (24.37)	379 (77.82)	
South	1,116 (37.91)	872 (78.08)		817 (37.61)	619 (77.02)	
**Body mass index (BMI)**			<0.001			<0.001
Underweight (<18.5)	37 (1.82)	28 (85.29)		30 (1.21)	20 (70.50)	
Normal weight (18.5–24.9)	780 (28.79)	661 (83.17)		577 (31.47)	488 (83.43)	
Overweight (25.0–29.9	909 (34.89)	765 (81.33)		597 (31.36)	489 (81.70)	
Obese (≥30.0)	927 (34.50)	624 (67.23)		694 (35.96)	473 (65.30)	
**Anxiety/depression symptoms**			<0.001			<0.001
None	1,820 (64.81)	1,495 (82.37)		1,295 (67.70)	1,053 (79.51)	
Mild	499 (19.89)	365 (72.03)		337 (17.94)	242 (73.37)	
Moderate	194 (7.95)	132 (66.16)		152 (8.07)	107 (74.41)	
Severe	140 (7.36)	86 (55.75)		114 (6.28)	68 (51.13)	
**Cigarette smoking status**			0.001			0.112
Never smoker	1,767 (94.60)	1,441 (79.36)		1,281 (65.33)	1,014 (78.73)	
Former smoker	542 (20.29)	412 (77.38)		373 (19.90)	289 (71.90)	
Current smoker	344 (13.34)	225 (65.20)		244 (14.77)	167 (70.87)	
**Cancer diagnosis status**			0.031			0.625
No	2,425 (94.60)	1,909 (77.77)		1,723 (94.20)	1,340 (76.35)	
Yes	228 (5.40)	169 (64.73)		175 (5.80)	130 (73.95)	

Data source: 2019 and 2020 Health Information National Trends Surveys, HINTS 5 Cycles 3 and 4, respectively. 2019–2020 unweighted n = 4,551 and weighted N = 325,250,679. Unweighted 2019 n = 2,653 and weighted N = 160,385,898. Unweighted 2020 n = 1,898 and weighted N = 164,864,782. Before the COVID-19 pandemic data (HINTS 5 Cycles 3) were collected from January through April 2019, and during the COVID-19 pandemic data were collected from February through June 2020. Frequencies were not weighted, while percentages were weighted.

## 3 Results

### 3.1 Prevalence of moderate intensity physical activity before and during the COVID-19 pandemic

Table 1 presents the prevalence of moderate intensity physical activity by sociodemographic characteristics and status of BMI, anxiety/depression, cigarette smoking, and cancer diagnosis before and during the COVID-19 pandemic. The prevalence of moderate intensity physical activity before the pandemic was higher (77.07%) than the prevalence during the pandemic (76.21%). Similar prevalence patterns were also observed within sociodemographic groups, BMI categories, anxiety/depression symptoms, cigarette smoking status, and cancer diagnosis status before and during the pandemic. However, the patterns were different between groups. For instance, the prevalence of moderate intensity physical activity decreased during the pandemic than before pandemic for age groups 26–34 and 50–64, with those aged 26–34 years having the highest prevalence: 26–34 (before = 84.42% vs. during = 83.66%) and 50–64 (before = 75.76% vs. during = 72.89%). The prevalence was lower before the pandemic among female than male population (before: female [76.66%] vs. male [77.45%]), but female individuals had higher prevalence than male individuals during the pandemic; during (female [76.37%] vs. male [76.06%]): the prevalence was marginally lower during the pandemic compared with before for both female and male individuals. For racial/ethnic groups, the prevalence had decreased for Hispanic (before = 75.61% vs. during = 71.93%), non-Hispanic Asian (before = 83.64% vs. during = 72.63%), and non-Hispanic other racial/ethnic group (before = 86.08% vs. during = 63.27%) populations but increased for non-Hispanic Black (before = 70.85% vs. during = 74.08%) and non-Hispanic White (before = 77.54% vs. during = 78.88%) populations. Lesbian, gay, and bisexual individuals (before = 85.23% vs. during = 75.24%) had a higher moderate intensity physical activity prevalence than their heterosexual counterparts (before = 76.63% vs. during = 76.28%), however, the prevalence had decreased for both groups.

### 3.2 The odds of engaging in moderate intensity physical activity before and during the pandemic

Before the COVID-19 pandemic, as shown in multivariable results (Table 2), the odds of engaging in moderate intensity physical activity were lower for individuals with fair or poor general health status (OR = 0.24, 95% CI = 0.27, 0.63) compared to their counterparts with excellent or good general health status. However, moderate intensity physical activity was not associated with general health status during the pandemic. Individuals who resided in the U.S. South had higher odds of engaging in moderate intensity physical activity (OR = 1.64, 95% CI = 1.05, 2.56) compared to those who resided in U.S. Northeast before the pandemic. The odds were higher also for those who resided in the U.S. Midwest (OR = 2.95, 95% CI = 1.63, 5.36), South (OR = 2.04, 95% CI = 1.19, 3.51), and West (OR = 1.92, 95% CI = 1.12, 3.31) compared to those who resided in U.S. Northeast during the pandemic. Those who were widowed, compared to single or never married individuals, had higher odds of engaging in moderate intensity physical activity during the pandemic (OR = 1.61, 95% CI = 1.05, 8.69), but there were no differences in the odds between all marital categories before the pandemic. Having less than High School education relative to college graduate or higher education was associated with lower odds of engaging in moderate intensity physical activity during the pandemic (OR = 0.33, 95% CI = 0.16, 0.70); the odds were lower for those who had family income of $20,000 - $34,999 (OR = 0.42, 95% CI = 0.24, 0.74) compared to those who had at least $75,000 annual income. Moderate intensity physical activity was not associated with level of education and annual family income before the pandemic. Individuals who were obese, compared to those who had normal weight or were underweight, had lower odds of engaging in moderate intensity physical activity before (OR = 0.51, 95% CI = 0.33, 0.79) and during (OR = 0.44, 95% CI = 0.28, 0.70) the pandemic. The odds were lower for individuals with mild, moderate, or severe anxiety/depression symptoms, compared to those with no/minimal symptoms of anxiety/depression, before (OR = 0.60, 95% CI = 0.42, 0.85) and during (OR = 0.61, 95% CI = 0.43, 0.86) the pandemic. No significant differences were observed in cancer diagnosis status during the pandemic, while individuals with a cancer diagnosis had lower odds of engaging in moderate intensity physical activity before the pandemic (OR = 0.56, 95% CI = 0.32, 0.98) compared to those without cancer diagnosis. Moderate intensity physical activity was not significantly associated with age, sex, race/ethnicity, sexual orientation, health insurance status, and cigarette smoking status before and during the pandemic.

**Table 2:** The odds of moderate intensity physical activity among U.S. adults aged 18–64 years before and during the COVID-19 pandemic, HINTS 2019–2020.

	**Before the COVID-19 pandemic (Model 1)**		**During the COVID-19 pandemic (Model 2)**	
**Characteristics**	**AOR**	**95% CI**	**AOR**	**95% CI**
**Age (Years)**				
18–25	Ref.	–	Ref.	–
26–34	1.76	(0.87, 3.57)	1.53	(0.64, 3.62)
35–49	1.03	(0.49, 2.14)	1.06	(0.53, 2.14)
50–64	1.23	(0.63, 2.40)	0.82	(0.42, 1.59)
**Sex**				
Male	Ref.	–	Ref.	–
Female	1.02	(0.73, 1.41)	1.00	(0.71, 1.40)
**Race/Ethnicity**				
Non-Hispanic White	Ref.	–	Ref.	–
Non-Hispanic Black	0.89	(0.50, 1.59)	1.11	(0.65, 1.90)
Hispanic	1.09	(0.72, 1.64)	0.91	(0.56, 1.49)
Non-Hispanic other	1.43	(0.79, 2.57)	0.60	(0.32, 1.12)
**Sexual orientation**				
Heterosexual	Ref.	–	Ref.	–
Lesbian/gay/bisexual	1.96	(0.89, 4.30)	1.06	(0.48, 2.38)
**Marital status**				
Single/never married	Ref.	–	Ref.	–
Married/living as married	0.95	(0.62, 1.45)	0.94	(0.57, 1.54)
Divorced/separated	1.01	(0.57, 1.77)	0.25	(0.51, 1.57)
Widowed	0.79	(0.30, 2.10)	1.61*	**(1.05, 8.69)**
**Level of education completed**				
College graduate/higher	Ref.	–	Ref.	–
Less than high school	0.61	(0.28, 1.35)	0.33**	**(0.16, 0.70)**
High school graduate	0.65	(0.41, 1.01)	0.68	(0.41, 1.12)
Some college	0.70	(0.49, 1.00)	0.73	(0.47, 1.14)
**Total family annual income**				
≥$75,000	Ref.	–	Ref.	–
<$20,000	0.78	(0.44, 1.36)	0.71	(0.36, 1.42)
$20,000 - $34,999	0.90	(0.48, 1.68)	0.42**	**(0.24, 0.74)**
$35,000 - $49,999	0.85	(0.49, 1.48)	0.67	(0.32, 1.40)
$50,000 - $74,999	1.09	(0.70, 1.69)	0.71	(0.40, 1.23)
**Health insurance status**				
Yes	Ref.	–	Ref.	–
No	0.69	(0.39, 1.21)	0.86	(0.46, 1.58)
**General health status**				
Excellent/very good/good	Ref	–	Ref	–
Fair/poor	0.42***	**(0.27, 0.63)**	0.75	(0.42, 1.34)
**U.S. census region**				
Northeast	Ref.	–	Ref.	–
Midwest	1.51	(0.92, 2.48)	2.95***	**(1.63, 5.36)**
South	1.64*	**(1.05, 2.56)**	2.04**	**(1.19, 3.51)**
West	1.14	(0.68, 1.90)	1.92**	**(1.12, 3.31)**
**Body mass index (BMI)**				
Normal weight/underweight	Ref	–	Ref	–
Overweight	0.98	(0.63, 1.52)	0.97	(0.57, 1.64)
Obese	0.51**	**(0.33, 0.79)**	0.44***	**(0.28, 0.70)**
**Anxiety/depression symptoms**				
None	Ref.	**–**	Ref.	–
Mild/moderate/severe	0.60**	**(0.42, 0.85)**	0.61**	**(0.43, 0.86)**
**Cigarette smoking status**				
Never smoker	Ref.	–	Ref.	–
Former smoker	1.02	(0.69, 1.51)	0.85	(0.52, 1.37)
Current smoker	0.65	(0.41, 1.03)	0.77	(0.42, 1.39)
**Cancer diagnosis status**				
No	Ref.		Ref.	–
Yes	0.56*	**(0.32, 0.98)**	0.92	(0.50, 1.68)

Data source: 2019 and 2020 Health Information National Trends Surveys, HINTS 5 Cycles 3 and 4, respectively. Before the COVID-19 pandemic data (HINTS 5 Cycles 3) were collected from January through April 2019, and during the COVID-19 pandemic data were collected from February through June 2020. AOR = Adjusted odds ratio. 95% CI = 95% confidence interval. Ref = Reference group. *p ≤ 0.05, **p ≤ 0.01, ***p ≤ 0.001.

## 4 Discussion

Moderate intensity physical activity was higher before the pandemic compared to during the pandemic, implying lower moderate intensity physical inactivity before the pandemic (22.93%) than during the pandemic (23.79%). Prevalence patterns also differed by sociodemographic characteristics and between groups. Our findings are important because they aid in understanding the pandemic’s impact on physical activity which has wide ranging public health ramifications. The physical activity guidelines for Americans recommend moderate intensity physical activity for 150 min weekly for individuals aged 18–64 (Piercy and Troiano, 2018). Moderate physical activity has been shown to be essential in mitigating several health conditions such as intermittent claudication, increased muscle strength, memory loss, blood glucose management, cardiovascular risks, and depression (Irwin et al., 2000, Sallis et al., 1986). Lin et al. also found that moderate physical activity is a modifying factor for depression during the pandemic (Lin et al., 2020). This finding is vital due to the widespread prevalence of obesity among all age groups in the general U.S. population and the potential role physical activity can play in reducing it. Therefore, it is imperative to understand and develop comprehensive strategies (e.g., home-based physical activity, telehealth services, transportation) aimed at improving moderate physical activity levels among the U.S. population, especially those with high morbidity and mortality risks.

We also found that the odds of engaging in moderate intensity physical activity before and during the pandemic were lower among obese individuals compared to non-obese individuals. Similarly, studies have shown that the obesity rates are likely to increase in the U.S (Ward et al., 2019, Finkelstein et al., 2012) and the pandemic’s impact on physical activity could further exacerbate this problem. There is a critical need to enhance physical activity to reduce and prevent obesity and other chronic medical conditions, which have been associated with increased morbidity and mortality risk from COVID-19 infections (Chen et al., 2022, Kompaniyets et al., 2021, Li et al., 2021, Kim et al., 2021). In particular, personalized physical activity interventions should be provided to those with fair/poor health, severe anxiety/depressive symptoms, a cancer diagnosis, or those currently smoking cigarettes to reduce further complications associated with their chronic and mental health conditions, as well as cigarette smoking. As the findings showed, individuals with mental health symptoms, fair/poor health, or those who engaged in cigarette smoking had a lower prevalence and odds of physical activity before and during the pandemic. One possible strategy for maintaining and improving physical activity during public health crises like the COVID-19 pandemic, while practicing social distancing or being quarantined, is a home-based intervention (Diniz et al., 2020, Emerenziani et al., 2018,, Gabriel and Zierath, 2019,). Home-based exercises (e.g., aerobic, stretching, and strength exercises) have been reported in intervention studies to improve cardiometabolic profiles, psychological wellbeing, and physical fitness in the general population and patients with cancer, kidney disease, and obesity (Diniz et al., 2020, Emerenziani et al., 2018,, Gabriel and Zierath, 2019,, Cheville et al., 2013, Tang et al., 2010).

Further, our findings revealed disparities in the prevalence of moderate intensity physical activity, with a disproportionately higher reduction observed among minority groups. This indicates the significant impact of the COVID-19 pandemic. Consistent with previous studies, we found lower prevalence rates of physical activity among certain demographic groups, including older adults, racial/ethnic minorities, sexual minority individuals (lesbian/gay, or bisexual), those with family income between $20,000 and $74,999, or uninsured individuals (Stockwell et al., 2021, Watson et al., 2021, Bu et al., 2021). The lower odds of physical activity among those with less than high school education and low income during the pandemic is consistent with previous studies before the pandemic (Scholes and Bann, 2007, Kari et al., Jul 2020, Droomers et al., 2001, Armstrong et al., 2018). Other studies have found increased mortality and morbidity rates among certain racial and ethnic groups, suggesting the need to enhance health-related behaviors like physical activity to reduce racial/ethnic health-related disparities among these populations (Habibdoust et al., 2022, Nguyen et al., 2022, Unruh et al., 2022, Grosicki et al., 2022, Walls et al., 2023). Furthermore, the significantly lower prevalence in moderate intensity physical activity behavior highlighted in this study among Black, Hispanic, and Asian individuals calls for greater efforts to encourage physical activity among these communities. Although the odds ratio estimates indicate lower likelihoods of engaging in physical activity among minority populations, the estimates were not statistically significant based on age, sex, race/ethnicity, and sexual identity before and during the pandemic. These findings suggest a potential lack of differences in physical activity based certain sociodemographic characteristics in this cross-sectional study, which requires further evaluation. Future longitudinal studies can help elucidate changes in or trajectories of physical activity based on the sociodemographic characteristics of the participants to inform interventions and policies on stability of physical activity during epidemiological crises. Future studies examining differences in moderate intensity physical activity behavior in the post pandemic era will be vital in aiding public health officials to design targeted educational strategies to enhance physical activity and its health benefits among diverse populations. Moreover, a detailed evaluation of the contributing factors of physical inactivity among individuals with low income and those with less than a high school education could help mitigate some of the health risks associated with inactivity and its impact on general health.

Individuals in the U.S. South were more likely to be active compared to those in the Northeast before pandemic. This may be due in part to the warmer climate in the southern U.S. during the 2019 data collection period from January through April 2019, which might have encouraged more outdoor physical activity despite the social distancing and stay-at-home policy. Higher odds were also observed during the pandemic in Midwest, South, and West compared to Northeast. In contrast, other studies have reported a higher prevalence and risk of physical inactivity in the South than in other regions of the U.S (National Center for Chronic Disease Prevention and Health Promotion, Abildso et al., 2023). The differences in the findings could be attributed to differences in physical activity measures, aged groups, and the data collection periods. Our study was conducted among only adults aged 18 to 64 years, while the other studies were conducted among all adults. Also, our study used HINTS data that were collected from January through April 2019 and February through June 2020, whereas the other studies either used the 2017–2020 Behavioral Risk Factor Surveillance System (BRFSS) combined dataset or the 2020 National Health Interview Survey (NHIS) data, which were collected throughout the year. Despite these differences in the findings, our findings and that of the other studies highlight the need for concerted public health initiatives aimed at improving physical activity in all regions of the U.S., especially in areas with high number of individuals with fair or poor health status. Additionally, understanding the disparities in this health-related behavior by geographic areas (region, state, county, or city) can help achieve CDC’s Active People, Healthy Nation, and Healthy People 2030 goal of examining and reducing geographic disparities in meeting guidelines (Abildso et al., 2023).

### 4.1 Limitations

The findings of this study should be interpreted with caution because HINTS dataset is a cross-sectional survey, as a result we are unable to establish causal relationship between physical activity changes before and during the COVID-19 pandemic. Also, the cross-sectional and self-reported nature of the data makes it susceptible to potential recall and underreporting biases. A single-item measure of physical activity intensity, which may not capture the full range of physical activity behaviors or intensity levels. More detailed measures of physical activity, such as accelerometry or daily activity logs, may provide a more accurate assessment of physical activity levels. Finally, the study did not examine the potential impact of the COVID-19 pandemic on other factors that may influence physical activity levels, such as access to recreational facilities or social support. These factors may be important to consider in future research on physical activity during the pandemic.

## 5 Conclusions

Moderate physical activity has been shown to be vital in the prevention of several chronic conditions and premature death. The ability to engage in physical activity (exercise) was significantly impacted by the stay-at-home order at the height of the declaration of the COVID-19 pandemic. The results of this study suggest that while the prevalence of moderate intensity physical activity remained high before and during the pandemic, there was a modest decrease in the prevalence during the pandemic, with certain subgroups, such as individuals with lower socioeconomic status and poorer mental and physical health, less likely to engage in moderate intensity physical activity. Given the direct relationship between physical activity, obesity, and high morbidity and mortality, it is important that public health officials, insurance providers, and policymakers collaborate locally to encourage physical activity in the post pandemic period. This study further underlines the need for concerted physical activity educational strategies aimed at addressing poor socioeconomic and mental and physical health conditions to improve access to and utilization of moderate intensity physical activity within population subgroups to reduce moderate intensity physical activity disparities, particularly among disadvantaged groups during pandemics. Future studies, including longitudinal studies, may evaluate the changes and trends in moderate intensity physical activity behavior within the population subgroups over time to determine the stability of the behavior for improved intervention development.

## Funding source

This work is supported by the Division of Intramural Research, National Institute on Minority Health and Health Disparities (ZIA MD000015). Opinions and comments expressed in this article belong to the authors and do not necessarily reflect those of the U.S. Government, Department of Health and Human Services, National Institutes of Health, and National Institute on Minority Health and Health Disparities.

## CRediT authorship contribution statement

**David Adzrago:** Conceptualization, Data curation, Formal analysis, Methodology, Validation, Visualization, Writing – original draft, Writing – review & editing. **Saanie Sulley:** Conceptualization, Methodology, Writing – original draft, Writing – review & editing. **Cameron K. Ormiston:** Writing – original draft, Writing – review & editing. **Faustine Williams:** Conceptualization, Methodology, Resources, Supervision, Visualization, Writing – review & editing.

## Declaration of competing interest

The authors declare that they have no known competing financial interests or personal relationships that could have appeared to influence the work reported in this paper.

## Data Availability

The HINTS data are publicly available at https://hints.cancer.gov/
